# Augmenting the National Nutrition Data System to Promote Diet Sustainability Analyses

**DOI:** 10.1016/j.cdnut.2024.103793

**Published:** 2024-06-06

**Authors:** Zach Conrad, Chloe DiStaso, Madison Korol, Donald Rose

**Affiliations:** 1 Department of Kinesiology, William & Mary, Williamsburg, VA, United States; 2 Global Research Institute, William & Mary, Williamsburg, VA, United States; 3 College of Arts & Sciences, William & Mary, Williamsburg, VA, United States; 4 Tulane Nutrition, School of Public Health and Tropical Medicine, Tulane University, New Orleans, LA, United States

**Keywords:** FCID, NHANES, sustainable, sustainability, database

## Abstract

Research on sustainable diets has become an important and growing area of the nutrition field, but recent studies have pointed to a lack of sustainability metrics and methods that are hindering research and policy progress. To fill this gap, the White House National Strategy on Hunger, Nutrition, and Health calls for increased funding to improve metrics, data collection, and research to address all domains of sustainability, which include nutrition/health, economic, environmental, and social domains. Commodity recipe databases, such as the Food Commodity Intake Database (FCID), are important tools for conducting diet sustainability analyses because they translate mixed dishes from dietary surveys, such as the National Health and Nutrition Examination Survey (NHANES), into commodity ingredients. These ingredients have been linked to data on environmental impacts and economic costs from other databases, thus facilitating collaboration between nutrition researchers, environmental scientists, economists, and others. These linkages cannot be made with other components of the national nutrition data system, such as the Food Patterns Equivalents Database (FPED), because the disaggregated food groups from them are not relevant for examining environmental impacts. Although the NHANES is conducted on an ongoing basis, and FPED is continually updated, the FCID has not been officially updated since 2010. This severely limits advancements in sustainability research and related policy analyses. In this commentary, we argue that the federal government should promote this diet sustainability work by integrating a commodity recipe database into the national nutrition data system, and updating it on a regular basis, as it does with other component databases.

## The Importance of Diet Sustainability Research

Poor diet quality is the leading modifiable risk factor for mortality in the United States, accounting for over 0.5 million deaths annually [1] and 45% of the cardiometabolic mortality burden [2]. Poor diet quality is also the third leading modifiable risk factor for morbidity, accounting for 11% of disability-adjusted life years [1]. Only 5% of the United States population meets dietary recommendations and disparities in diet quality have worsened across income groups [3,4]. Suboptimal diet quality also accounts for 18% ($50 billion) of total direct medical costs in the United States, and individual costs are higher for lower-income minority groups [5] which are often unable to afford the higher monetary costs of healthier diets [6,7]. Federal investment in nutrition research has been inadequate to meet this challenge [8]. For example, the National Institutes of Health is the largest funder of nutrition research but only 5% of its budget is allocated for this purpose [8].

At the same time, diet patterns are a leading driver of environmental impacts that can no longer be ignored. United States diet patterns account for nearly 20% of national greenhouse gas emissions [9] and per capita emissions are over 70% higher than the global average [10]. The United States food system also accounts for over one-quarter of freshwater withdrawals and land use, and over 10% of fossil fuel use [9]. Concerns about safe working environments are also emerging [11], as well as other indicators of social sustainability such as food availability and cultural acceptability [12]. These impacts vary tremendously by which commodities are produced for human consumption [11,13,14] and dietary shifts are urgently needed to bring the United States and global food systems within planetary boundaries [15,16].

To inform sustainable policy agendas, more research is needed to evaluate the impact of diet patterns on all domains of sustainability so as to better understand synergies and trade-offs between these domains, which can help avoid unintended consequences. These are known as diet sustainability analyses. For example, recent studies have demonstrated that simple dietary substitutions can lead to substantially reduced environmental impacts [17,18], and have demonstrated the trade-offs between increasing different components of diet quality and minimizing environmental impacts and monetary costs [[19], [20], [21]].

Yet recent studies have pointed to a lack of sustainability metrics and methods that are hindering further research and policy progress [12,22,23]. The National Strategy on Hunger, Nutrition, and Health (hereafter, the National Strategy), released by the White House in September 2022, aims to fill this gap. Divided into different themes or “pillars,” “Pillar 5-Enhance Nutrition and Food Security Research,” calls for improvements to nutrition data metrics, data collection, and research to inform nutrition and food security policy, particularly on issues of equity, access, and disparities [24]. For example, it calls for a new iteration of the National Household Food Acquisition and Purchase Survey (FoodAPS) [25], last released in 2013, which provides novel information on community food environments and food spending. The FoodAPS has been used to develop new metrics used to measure consumer spending on foods purchased for consumption away from home [6,19,26,27], which represent over 50% of total food spending [28].

**FIGURE 1:**
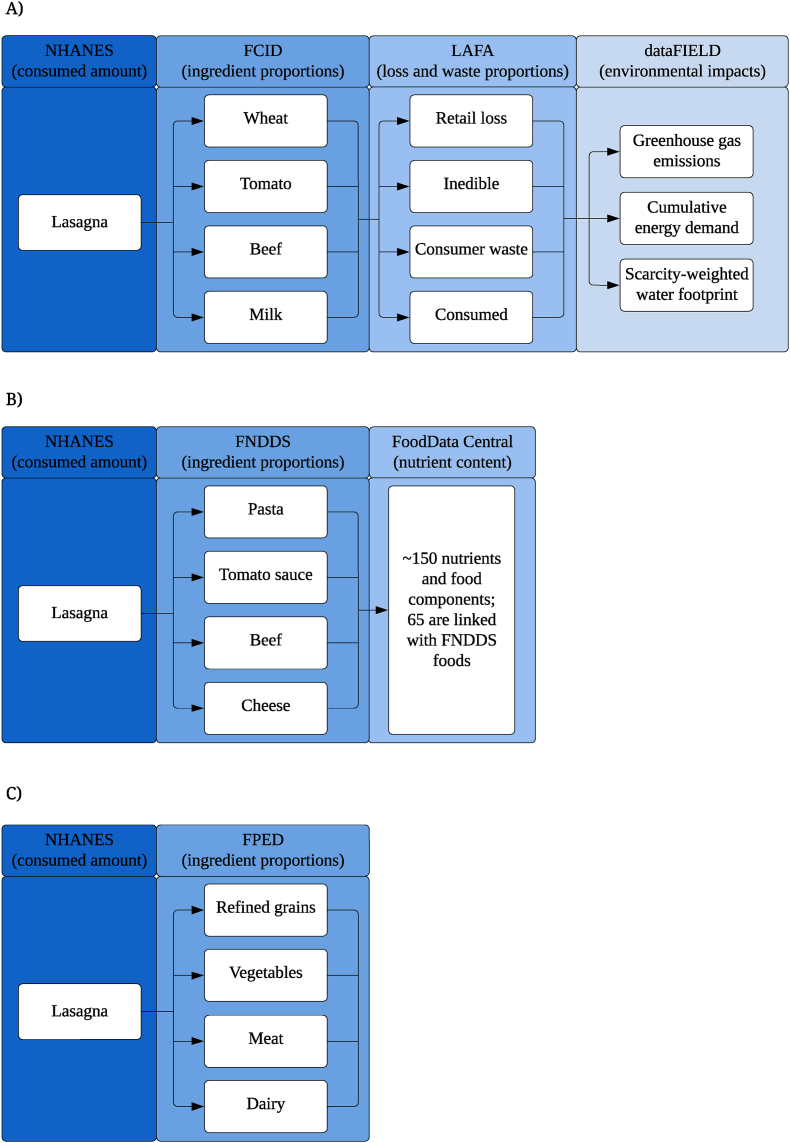
Database linkage structure for (A) the Food Commodity Intake Database, (B) the Food and Nutrient Database for Dietary Studies, and (C) the Food Patterns Equivalents Database. FCID, Food Commodity Intake Database; FNDDS, Food and Nutrient Database for Dietary Studies; FPED, Food Patterns Equivalents Database.

This same effort should be applied to updating other important databases, particularly the Food Commodity Intake Database (FCID) [29], which provides the data infrastructure needed to perform many types of diet sustainability analyses [6,13,[19], [20], [21],^26^,^27^,[30], [31], [32], [33]]. The FCID provides recipes that disaggregate mixed dishes from national dietary surveys into commodity ingredients (agricultural products), which can then be linked to sustainability data from other databases (Figure 1). Compared with other food and commodity composition databases, the data infrastructure of the FCID is more compatible with linkage to sustainability databases, is easier to maintain on a regular basis, and is the most commonly used for diet sustainability studies that use dietary data from the National Health and Nutrition Examination Survey (NHANES), which is the richest source of nationally representative dietary data in the United States and is the principal source of information for developing nutrition policy [34,35]. However, the FCID has not been officially updated since 2010 which severely limits advancements in sustainability research that are needed to answer important policy questions. For example, there is a need to estimate the sustainability impacts of nationally representative diet patterns at regular intervals to evaluate progress toward meeting national sustainability objectives, which is hampered without regular updates to the FCID. Important types of nutrition research are also at risk without timely updates to the FCID, particularly those that investigate the link between food consumption and human exposure to pesticides. The National Strategy provides the critical impetus to fill this gap by investing in regular updates to this important database.

## A National Nutrition Data System

Although there is no officially designated “national nutrition data system” in the United States, the country has a rich set of data sources that enable monitoring and research on many aspects of nutritional health. The resources of this de facto system have been well documented over the years, beginning with the seminal report, *Nutrition Monitoring in the United States: The Directory of Federal and State Nutrition Monitoring and Related Research Activities*, an interagency effort distributed by the Centers for Disease Control and Prevention [36]. A more recent catalog of surveillance systems, focused toward child nutritional health, has been collated by the National Collaborative on Childhood Obesity Research [37]. Clearly at the center of such a national system are the ongoing surveys and data sources that record food intake data of the population, such as the NHANES. Also at the center of this system are the food composition databases that enable researchers to translate what survey respondents report eating into nutrient intakes and food groups, thus allowing for evaluation of their diets.

Beginning in 2019, 5 of these databases were aggregated into FoodData Central (FDC), maintained by the USDA Agricultural Research Service (ARS) [38]. The Food and Nutrient Database for Dietary Studies (FNDDS) [39], 1 of these 5 databases, is the key source for linking foods from NHANES survey data to their nutrient contents. For example, FNDDS can be used to estimate the amount of pasta (a processed food) in a lasagna dish, which can then be linked to the nutrient profile of pasta from other FDC databases (Figure 1). For a given nutrient of interest, the nutrient content of all other ingredients in all other mixed dishes can be summed for each study participant, which can be used to evaluate total nutrient intake among the general population. The FPED, also maintained by USDA ARS, allows the translation of foods reported by NHANES respondents into the nutritionally-oriented food groups used to assess diet quality and adherence to the Dietary Guidelines for Americans (DGA). For example, the FPED can be used to estimate the amount of refined grains (a food group) in lasagna, which is a variable used to calculate diet quality using the Healthy Eating Index-2020. Both of these databases (FNDDS and FPED) are updated every 2 y as new waves of NHANES are fielded. This same kind of regularity with a commodity-based database, such as FCID, would allow for ongoing diet sustainability analyses, as we discuss in the next section. For example, the FCID can be used to estimate the amount of wheat (an agricultural commodity) in lasagna, which can be linked to data on food waste, greenhouse gas emissions, and other environmental impacts from other databases. The 3 databases—FNDDS, FPED, and FCID – can all be linked to consumer diets in NHANES, but fulfill different purposes, reflective of their different entry points in the food system. FCID connects data at the agricultural level, that is, commodities such as wheat, which allows for use by agricultural and environmental scientists, who also work with commodity data. FNDDS connects data from the food processor level, the ingredients of mixed dishes, such as pasta, that allows for understanding the nutrient composition of mixed dishes. FPED regroups the foods eaten by consumers into nutritionally-oriented food groups, such as refined grains. See the subsequent section for additional discussion of FDC, FNDDS, FPED, and the limitations of these and other federal databases for sustainability research.

## A Missing Ingredient to Promote Diet Sustainability Analyses: the FCID

Diet sustainability analyses rely on food and commodity composition databases to translate mixed dishes reported by diet survey respondents into their constituent ingredients [40], which can then be linked to databases on environmental impacts, food prices, and pesticide exposure. For the FCID, these constituent ingredients are commodities, which are agricultural products such as wheat and tomatoes, rather than processed food products that make up the mixed dishes consumers eat, such as pasta and tomato sauce. Commodities are key variables in many other disciplines, thus enabling interdisciplinary research between nutrition science, crop and marketing science, environmental science, economics, and social science (Figure 1). For example, crop and marketing scientists use commodity information to assess the consumer demand for the foods they study, and environmental scientists conduct their studies at the level of the commodity. Connecting these impacts to dietary intakes through a database like the FCID allows for assessing the carbon footprint of a diet, for example. The sections below review the empirical work in diet sustainability that has been made possible by the FCID.

### Nutrition and health

The FCID has been used to estimate exposure to a wide range of chemicals and compounds such as food additives [41], metals [42,43], bisphenol A [44], foodborne pathogens [[45], [46], [47]], and others [[48], [49], [50], [51], [52], [53], [54], [55], [56]]. Because FCID is the only food composition database that disaggregates mixed dishes into commodity-specific foods, others have used it to estimate the consumption of mushrooms [[57], [58], [59], [60]], rice [53,54,[61], [62], [63], [64]], multiple types of lentils and legumes [[45], [46], [47],^65^], beef [[66], [67], [68], [69], [70]], pork [66,67,69,70], poultry [51,52,66,67,69,70], seafood [67,69,70], multiple types of plant oils [71,72], water [73], and others [74]; as well as mixed dishes such as pizza [75]; and food groups such as dairy [69,70], fruit [76], vegetables [76], and nuts and seeds [69,70,77,78].

The FCID has also been used to estimate the contribution of foods to nutrient intake, including fatty acids [68,71,72,79], saccharides [[80], [81], [82], [83]], protein [69,70], and others [60,[62], [63], [64],^78^,^84^]. When combined with data on health status, FCID has been used in studies to evaluate the association between dietary intakes and health markers such as cancer [76], depression [58], cognitive function [77], adiposity [81], serum lipids [64,81], inflammatory markers [64], blood pressure [81], liver disease [77], mortality [57,59,85], and others [65,[80], [81], [82]].

### Environment

The FCID has been linked to several types of computational models that have been used to evaluate the environmental impacts of dietary intake. The FCID has been linked to data from life cycle analysis (LCA) [13,32,75,86] to evaluate the association between dietary intake and greenhouse gas emissions [13,19,21,[31], [32], [33],^75^,[86], [87], [88], [89], [90], [91]], energy use [13], water use and scarcity [32,86,90,91], and land use [86,90,91]. Greenhouse gas emissions have also been evaluated using carbon accounting methods [92]. Others have used the FCID in conjunction with footprint models to evaluate the agricultural resource demand of dietary intakes, including the use of land, fertilizer nutrients, pesticides, and irrigation water [20,30]. At the core of all these analyses is the translation of as-consumed foods into commodities. Nutrition surveys collect information from individuals about diets composed of mixed dishes, like lasagna, but environmental scientists use life cycle assessments and other computational models to analyze the impacts of specific commodities like tomatoes. The FCID allows the linkage of these 2 fields by translating lasagna into its “commodity ingredients” (that is, grams of tomatoes).

Given its versatility, prior studies have used the FCID to evaluate the environmental impacts of individual foods or food groups [13,20,21,30,32,86,88,90,91] as well as diet patterns, including actual [19,31,88] and modeled [87] diet patterns. These also include diet patterns differentiated by diet quality [19,20,30,31,33,88] or other characteristics such as meat intake [87], as well as counterfactual scenarios such as adherence to dietary recommendations [90,93] or food substitutions [33]. Others have used the FCID to evaluate the environmental impacts of food loss and waste [13,[19], [20], [21],^30^,^87^,^88^] as well as the impacts associated with demographic and behavioral characteristics [21,86,[89], [90], [91], [92]].

### Economic

To facilitate the analysis of individual-level diet costs, the USDA Center for Nutrition Policy and Promotion (CNPP) and Economic Research Service (ERS) developed the Food Prices Database, which provides the price per gram of each food in NHANES 2001–2004 [94]. More recently, the USDA ERS released the Purchase-to-Plate Price Tool which provides the price per gram of each food in NHANES 2011–2018 [95]. However, these databases only provide the price of the consumed portion of food rather than the amount that will eventually be wasted and inedible and therefore do not represent the total purchased price of food. To fill this gap, others have linked data on commodity level food loss and waste from the USDA loss-adjusted food availability (LAFA) data system to the NHANES by using FCID to bridge these databases [26]. Because the USDA LAFA provides loss and waste estimates at the commodity level, and the FCID links these commodities to mixed dishes in NHANES, the FCID is needed for this type of economic analysis. This method has been used to evaluate consumer spending on food at home and food away from home [6,19,26,88], the relationship between diet cost and diet quality [6,88], and the cost of popular diet patterns [19,88].

### Social

Social sustainability is concerned with measuring impacts on people, such as equity, social well-being, ethics, inter-individual interactions, and societal functions [96], and remains 1 of the most understudied domains of sustainability [12]. This domain can be measured using exposure metrics such as demographic and behavioral characteristics as well as outcome metrics such as forced labor [97] and animal welfare [98]. Demographic and behavioral characteristics are important exposure variables that can provide information on disparities in outcomes such as food access, food availability, and social risk that would otherwise be hidden by population averages [12]. Others have used FCID in conjunction with NHANES and other dietary surveys to evaluate these exposure metrics, including differences in food and nutrient intake by demographic characteristics such as age, sex, income, race, ethnicity, educational attainment, and others [59,60,62,64,65,[68], [69], [70],[71], [72], [73], [74],^82^,^84^], as well as behavioral characteristics such as smoking, exercise, and diet knowledge and behaviors [62,66]. The FCID has also been used to evaluate sociodemographic disparities in pesticide and chemical exposure [43,44,51,[53], [54], [55],^99^] and environmental impacts [21,86,[89], [90], [91], [92]].

## Not all Food Composition Databases are Equally Useful for Diet Sustainability Analyses

### FDC and the FNDDS

FDC was launched in 2019 by the ARS at the USDA and represents a compilation of 3 existing databases (FNDDS, USDA National Nutrient Database for Standard Reference, and USDA Global Branded Food Products Database) and 2 novel databases (Foundation Foods and Experimental Foods) [100]. USDA National Nutrient Database for Standard Reference and USDA Global Branded Food Products Database provide data on the nutrient content for >8000 foods, and ∼3000 of these foods are combined in various recipes to represent the 4500 foods and mixed dishes in the NHANES [101]. This linkage is facilitated by the food composition database FNNDS [39].

Foundation foods provides metadata for a portion of these foods, with plans to extend coverage to all foods as the data becomes available [100]. Some of these metadata, such as agricultural production practices, could be potentially valuable for some types of diet sustainability analyses, but they are currently unavailable and it is not clear when they will be released and what the data coverage will be. Furthermore, there is no indication that Foundation Foods will include quantitative data on sustainability indicators that are most impactful for policy action and most commonly used in sustainability studies, such as greenhouse gas emissions, energy use, water use, food waste, and food prices. Experimental Foods will provide data for foods produced using novel methods that may not be commercially available, such as foods produced using alternative agricultural management systems and experimental genotypes. These data could include information on genetics, environmental inputs and outputs, supply chains, and economics [102], which could potentially be useful for some types of diet sustainability analyses. However, this database is not currently available and data on noncommercial foods are of no value for population studies that evaluate the sustainability impacts of foods reported in dietary surveys, such as NHANES.

A more serious limitation with FDC, and with FNDDS in particular, is that given its data structure, it is likely not possible and certainly not cost effective to link all 8000+ foods with sustainability impacts. Environmental scientists work at the level of the commodity, studying the impacts of producing a kg of wheat, for example. To reliably quantify a given sustainability impact (for example, kg of greenhouse gas emissions per gram of food consumed) for a given food (for example, chicken meat), it may require many data points that are acquired from a comprehensive review of the literature. Such data are simply not available for most mixed dishes. Even using FCID, which translates FNDDS mixed dishes to commodities, and an exhaustive search of 12 y of environmental impact literature, Heller et al. [13] needed to use many substitutions to match impacts to commodity foods that did not have environmental impact studies.

### Food Patterns Equivalents Database (FPED)

The FPED is maintained by the Food Surveys Research Group at USDA ARS. The FPED converts each of the foods and mixed dishes in NHANES into 10 food groups and 27 subgroups (in servings) that align with the DGA [103]. These broad food categories are useful for evaluating adherence to dietary recommendations but are too broad to account for important differences in the sustainability impacts of different foods within the same category. For example, the FPED does not differentiate beef from pork (both are included in the “meat” category), which is associated with a 6-fold difference in greenhouse gas emissions per kg of food [104]. Given that these foods are consumed in different quantities, it is important to account for their different sustainability impacts at the food level to more accurately estimate these impacts at the person level and population level. These differences are particularly important to consider when performing food substitution analyses, such as substituting beef for pork or other protein foods [17].

## Challenges and Opportunities

The FCID is a highly versatile research tool that is regularly used to evaluate all domains of sustainability, but it remains sorely outdated which limits further sustainability research. Others have demonstrated that using outdated data from FCID can severely bias estimates of daily food intake [105]. The National Strategy provides the impetus for the federal government to resume regular maintenance of this valuable database, just as it recommended another iteration of FoodAPS [24].

The FCID should be linked to foods in the FNDDS 2011–2020 [39], which includes all of the foods in NHANES 2011–2020. Although others have linked the FCID to the FNDDS 2011–2018 [105,106], regular updates to federal databases should fall within the purview of the federal government rather than individual research teams at universities. The USDA Food Surveys Research Group is responsible for maintaining other food and nutrition databases (for example, FNDDS and FPED) and may be able to support regular maintenance of the FCID as well. These updates should account for product reformulations that have occurred since the last update, which occurred in 2010. Thousands of new and reformulated food products have entered the United States market each year since 2010 [107], and this is reflected in FNNDS which includes ≤1100 new foods and ≤1800 discontinued foods in each 2-y version [108].

Finally, regular updates to the FCID should be part of a larger coordinated effort by the federal government to support diet sustainability research through improved metrics and data collection programs [8,24]. For example, interagency efforts could be undertaken to promote the further study of environmental impacts of FCID commodities, so as to optimize the utility of updates. The Dutch National Institute for Public Health and the Environment has done this, providing dietary data and linked environmental LCA data that facilitates diet sustainability research. This approach can be applied to the United States setting and would benefit the work of several federal agencies. The Environmental Protection Agency could benefit in its mission to regulate pesticide use by a better understanding of consumer exposure to new pesticides [109], as well as their work on documenting the sources of greenhouse gas emissions [110]. The Food and Nutrition Service would be able to better inform consumers about sustainable diets, as would the ERS, in terms of the costs of such diets. Collating this literature into usable databases on a regular basis, as others have done earlier [13], would make it easier for nutrition researchers to incorporate sustainability analyses into their work. Future data collection efforts could enhance NHANES to better understand consumer attitudes about dietary change to promote environmental sustainability. Going forward, it will be important that additional responsibilities by the agencies in this area will be supported by additional budgetary resources.

## Author contributions

The authors’ responsibilities were as follows – ZC, DR: designed the study; ZC, CD, MK: conducted the research; ZC, DR: wrote the article; ZC: has primary responsibility for final content; and all authors: read and approved the final manuscript.

## Conflict of interest

ZC has research awards from the USDA and the Jeffress Trust Awards Program in Health Equity Research for projects unrelated to the present study. DR has research awards from the USDA and the Department of Health and Human Services for projects unrelated to the present study.

## Funding

This work was supported by the Margaret S. Glauber Faculty-Student Research Fellowship, the Office of the Vice Provost, and the Commonwealth Center for Energy and the Environment at William & Mary. The funders had no role in the design, implementation, analysis, or interpretation of the data.
